# The One Health aspect of climate events with impact on foodborne pathogens transmission

**DOI:** 10.1016/j.onehlt.2024.100926

**Published:** 2024-11-03

**Authors:** Igori Balta, Joanne Lemon, Ciara Murnane, Ioan Pet, Teodor Vintila, David McCleery, Todd Callaway, Alastair Douglas, Lavinia Stef, Nicolae Corcionivoschi

**Affiliations:** a Faculty of Bioengineering of Animal Resources, University of Life Sciences King Mihai I from Timisoara, 300645 Timisoara, Romania; b Chief Scientific Adviser's Office, Department of Agriculture, Environment and Rural Affairs for Northern Ireland, Belfast BT3 9ED, Northern Ireland, UK; c Trinity College Dublin, School of Medicine – Physiotherapy, College Green, Dublin 2, Ireland; d Bacteriology Branch, Veterinary Sciences Division, Agri-Food and Biosciences Institute, Belfast BT4 3SD, Northern Ireland, UK; e Department of Animal and Dairy Science, University of Georgia, Athens, GA, USA; f Veterinary Sciences Division, Agri-Food and Biosciences Institute, Belfast BT4 3SD, Northern Ireland, UK; g Academy of Romanian Scientists, Ilfov Street, No. 3, 050044 Bucharest, Romania

**Keywords:** One Health, Foodborne pathogens, Climate change, Climate events

## Abstract

The ongoing effects of climate change have exacerbated two significant challenges to global populations: the transmission of foodborne pathogens and antimicrobial resistance (AMR) through the food chain. Using the latest available scientific information this review explores how climate-related factors such as rainfall, floods, storms, hurricanes, cyclones, dust, temperature and humidity impact the spread of the foodborne pathogens *Salmonella*, *E. coli*, *Campylobacter*, *Vibrio*, *Listeria*, and *Staphylococcus aureus*. We explore the complex dynamics between environmental changes and the heightened risk of foodborne diseases, analysing the contribution of wildlife, insects and contaminated environments in the proliferation of AMR and climate change. This review paper combines a thorough analysis of current literature with a discussion on findings from a wide variety of studies to provide a comprehensive overview of how climatic factors contribute to the survival, persistence and transmission of bacterial pathogens in the food chain. In addition, we discuss the necessity for effective mitigation strategies and policies. By providing insights into the interrelationships between climate change and food safety, this review hopes to inform future research and policy development to promote safer and more sustainable food systems and further integration within the One Health approach.

## 1 Introduction

Over one hundred zoonotic pathogens have been identified as impacting humans through the food chain [1]. Despite this abundance, most food-related human disease can be traced back to just a few species. Climate change increasingly influences environmental contamination. Higher temperatures have the potential to boost the survival and presence of disease-associated bacteria in the environment and heavy rainfall events augment the spread of bacteria from farms and sewage systems into river catchments [2]. The importance of these climate factors cannot be overstated when developing future mitigation strategies and infrastructure to address the effects of climatic changes on public health. In recent decades, the changes in the production of food, processing and distribution have heightened the risk of foodborne diseases creating new opportunities for pathogen transmission to humans, specifically zoonotic pathogens like *Salmonella*, *E. coli*, *Vibrio* spp. and *Campylobacter* [3]. The global populations increase in demand for food has led to the expansion of industrial production systems, including the intensification of animal production, agriculture, large-scale food processing, catering, and distribution [3]. We will review documented studies and climate models, which are useful tools for simulating and predicting future climatic conditions and their impact upon agriculture, prices, delivery, quality, and food safety [4]. The global approach to food supply has already reshaped food consumption patterns and climate change is will further shift food production lines, stemming in a more global and diverse food supply for consumers [4].

**Fig. 1:**
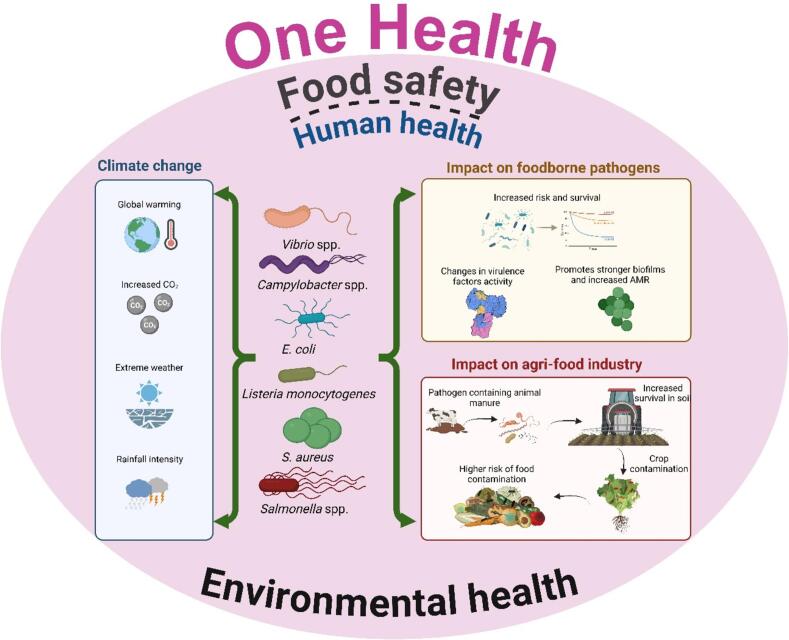
The impact of climate events on *Vibrio* spp., *Campylobacter* spp., *Listeria monocytogens*, *S. aureus* and *Salmonella* spp., within the One Health approach to climate change and impact on agri-food industry.

As climate change continues to alter environmental conditions, understanding the impact on foodborne disease transmission becomes increasingly critical. As displayed in Fig. 1, this review explores the complex interplay between climate change, specific pollutants (antibiotics, microplastics, and heavy metals), the transmission of bacterial pathogens and antimicrobial resistance (AMR) within the food chain. By examining the influence of major environmental factors such as water (rains, floods, storms), air (hurricanes, cyclones, dust), temperature, and humidity, the review seeks to understand how these climatic changes facilitate the spread of zoonotic pathogens like *Salmonella*, *E. coli*, *Campylobacter*, *Vibrio*, *Listeria*, and *Staphylococcus aureus*. Furthermore, the review will address the role of wildlife, insects and environmental contamination in the proliferation of AMR, providing insights into potential mitigation strategies to enhance public health and food safety in the context of a changing climate. (See Table 1).

**Table 1:** Climate events with impact on foodborne pathogen spread and transmission.

Climate events	Impact	Reference
Floods and rainfalls	- promote pathogen transmission (*Listeria* spp.) - Spread of *Salmonella* spp., from rooftops into soil (mainly from faecal droppings) - Increased water turbidity linked to increased gastrointestinal illnesses - Contributes to the transmission of pathogens such as *Aeromonas* spp., *Campylobacter* spp., *Salmonella* spp., and *Listeria* spp., as well as *E. coli* and *Legionella* spp. - Significant impact on aquaculture industry through the dissemination of antibiotic resistant bacteria	[5] [6] [7] [8] [7] [9]
Surface and waste waters	- Harbour pathogenic microorganisms of faecal origin - Increased rainfall due to climate change affects the transfer, prevalence and persistence of pathogens in agricultural water and on crops (e.g. *C. jejuni*, *Salmonella*). - Using surface waters for irrigation purposes has the potential to contaminate fresh produce with antibiotic-resistant bacteria (ARB) - The utilisation of untreated wastewater increases the transmission of pathogens to fresh produce intended for human consumption - Can promote the transmission of antibiotic-resistance genes (ARGs) and antibiotic-resistant bacteria (ARB)	[7] [7] [9] [7] [9]
Foodborne Aerosolised Microorganisms and their Dispersal	- Aerosolized microorganisms affect the terrestrial and aquatic microbiota and plays a major role in climate change related bacterial threats through soil, water pollution, and ultimately disease transmission - Projected drier and warmer summers will increase soil evaporation, encouraging the release of dust into the atmosphere - Dust storms contribute to the dispersion of microorganisms and bacterial levels from atmospheric samples were found to be increased during dust storms compared to background levels - Airborne dust can carry up to 27 times more bacteria than clean air, with total bacterial cell concentrations in airborne dust ranging between 10^6^ and 10^7^ cells/m^3^. - Soil dust serves as a unique pathway for transporting pathogens, such as foodborne and zoonotic bacteria (e.g., *E. coli, Salmonella*, *C. perfringens*, *Staphylococcus* and *Campylobacter* spp.), fungal and bacterial spores, including viruses through the air - Bacteria in dust samples will likely have greater survival rates if resistant to multiple environmental stresses - tropical cyclones, hurricanes and subsequent flooding events are linked to outbreaks of foodborne and waterborne diseases	[6] [10] [11] [12] [10,11]. [11,12] [11].

## 2 Impact of climate events on the growing threat of specific foodborne pathogens

### 2.1 *Vibrio* spp

*Vibrio* spp., a bacterium commonly found in marine and estuarine waters worldwide, is particularly concerning, because previous similar incursions were only observed in tropical and subtropical locations, which are now changing due to climate dynamics [13]. In shellfish, *Vibrio* spp., presence is highly dependent on seawater temperature, with studies demonstrating enhanced *Vibrio* spp. loads in aquatic environments with increased temperatures, resulting in more frequent disease outbreaks [13,14].

Rising water temperatures, as a consequence of global warming, promotes the growth of *Vibrio* spp. with studies showing a water temperature above 12 °C and with low to moderate salinity (1–25 g/L) to be optimal [15]. This paper demonstrates that global warming is undeniably contributing to the spread and higher prevalence of *Vibrio* spp., and to an increased risk of human infections rates in coastline areas and estuaries [15]. The permanent rise in water temperatures will likely amplify *Vibrio* contamination in the European fishing and farming sectors, extending beyond the summer and autumn. Over the last 30 years, heatwaves in northern Europe have been linked to an increase in *Vibrio* soft tissue infections. Incidences of these bacterial pathogens are particularly alarming in regions with elevated water temperatures, especially in the Southern Hemisphere, while the occurrence rates in several Northern Hemisphere countries are also rising due to water acidification, increasing temperatures and altered salinity [16]. In 2014, the highest number of annual *Vibrio*-associated soft tissue infections reported in Sweden and Finland coincided with the region's most severe heatwave [17]. In Denmark, human *Vibrio* infections have been steadily increasing since 2016, [16] from 26 in 2017 to 60 in 2018 and 69 in 2019 and are in correlation with high summer coast line water temperatures and low salinity [13]. These were primarily involving the species *V. cholerae* and *V. parahaemolyticus* [16]. Recent findings suggest that high pH and elevated temperatures in the Belgian regions of Wallonia, Flanders and the North Sea are favourable factors for the growth of *Vibrio* spp., specifically *V. alginolyticus* and *V. parahaemolyticus* [14].

In addition to increased temperatures, several climatic conditions such as precipitation, salinity and wind speed influence the growth and transmission of *Vibrio* spp. [18]. A positive correlation has been observed between the frequency of infectious *Vibrio* spp., including *V. vulnificus*, *Vibrio* cercariae, *V. parahaemolyticus*, and *V. alginolyticus* in coastal waters and turbidity or recent heavy rainfall [18]. In relation to turbidity, a field study in the Florida Keys showed that the growth of total *Vibrio* spp. intensified with the addition of dust to seawater [18] and the same study conducted in synthetic iron-diminished seawater showed that the growth of *V. alginolyticus* and *V. cholerae* increased when dust was added to the medium [18].

### 2.2 *Campylobacter* spp

Approximately 30 % of all human infections are attributed to poultry consumption, 20–30 % from beef and a smaller percentage of infections arise due to pathogenic strains from other sources, including game animals [19]. *Campylobacter* levels in chicken flocks fluctuate with temperature and humidity, in contrast human cases are linked to fluctuations in temperature and precipitation, particularly during extreme precipitation events [20]. Seasonality significantly influences *Campylobacter* spread and contamination, with a statistical correlation observed between temperature and contamination levels [21]. Using Ontario as an example, an exploratory study revealed a positive correlation between climate and bacterial prevalence [21]. The knowledge that *Campylobacter* cannot replicate outside the host implies that the hotter temperatures attributed to climate change alter people's behaviour leading to riskier food consumption patterns, rather than the inherent replication capability of the bacteria [22].

In contrast, rainfall and wind speed have a lesser influence on the incidence of Campylobacteriosis [13]. In Europe, campylobacteriosis cases are clearly associated with changes in temperature rather than precipitation [13]. As the association with temperature was not direct, the relationship is probable indirect and might be influenced by other seasonal mechanical vectors, for instance, fly transmission [22]. Studies using national surveillance data to analyse the association between climate and campylobacteriosis in Denmark, Finland, Norway and Sweden have revealed that *Campylobacter* incidences are related to rises in temperature and in particular precipitation in the week preceding illness, proposing a non-food related spread route for the disease [20]. To concur with this, their models indicated that heat waves and winter precipitation (rain and snowfall) may reduce the total of reported cases. The past eight years have been the hottest on record, with 2022 seeing the total 10-year average temperature rise to 1.15 °C above preindustrial levels [23].If this trend continues, a recent study forecast an almost 200 % increase in campylobacteriosis in Scandinavian countries by the end of the century, which could result in nearly 6000 additional cases per year [22,23]. This rise is attributed to an extended transmission season and other climatic changes.

### 2.3 *Escherichia coli*

As ambient temperatures rise, the proliferation of *E. coli* in the environment and food products increases, elevating the risk of contamination and infection. For instance, EHEC O157:H7, notorious for its severe outbreaks often linked to undercooked meat and raw vegetables, can thrive in warmer conditions thus amplifying its transmission during heat waves [24]. In a study across the EU and EEA between 2015 and 2019, Gilligham et al observed that confirmed cases of STEC rose during the months April and May and peaked from June to September [13]. In England, from 2015 to 2019, STEC cases increased from April until July (in 2016) or August (in other years) before declining [13,25]. Moreover, in 2019, the highest incidence rate in the EU and EEA was among children aged 0 to 4 years, who comprised over a quarter of all confirmed cases, while the lowest incidence was between individuals aged 45 to 64 years.

A recent laboratory investigation showed that higher temperatures correlate with increased proliferation of *E. coli* in American oysters (*Crassostrea virginica*), a key seafood species [26,27]. Using oysters found in the southern waters of the Gulf of Mexico, this study demonstrated that *E. coli* proliferation escalated as water surface temperatures rose from 24 to 32 °C [26]. An experimental French study observed that a similar correlation between temperature and microbial growth occurred with unpasteurised milk [28]. The model predicted that warmer temperatures, combined with delays along the supply chain, would lead to greater concentrations of *E. coli* in milk, potentially increasing the infection risk from unpasteurised milk consumption in the future [28]. Furthermore, climate change is expected to have both positive and negative effects on the persistence of STEC O157 in manure, soil and water, which could consequently affect contamination levels in leafy green vegetables [28].

The frequency of *E. coli* O157:H7 infections in humans and the shedding of VTEC by cattle are known to exhibit seasonality [29]. This seasonality is partly attributed to the enhanced survival and proliferation of *E. coli* O157:H7 in faeces at higher temperatures [29]. *E. coli* O157 has been linked to pre-harvested leafy green vegetables. STEC grows optimally at 37 °C but can grow in temperatures varying from 7 °C to 50 °C [13]. A study on the level of STEC infections in children from Italy observed an increase in the frequency of cases during heatwaves and cases were of longer duration and greater magnitude [30]. Given the increased probability of more frequent heatwaves in the future, it is probable that the number of STEC infection cases will also rise [30] as warm ambient temperatures were recently linked to increased shedding of STEC in wild deer [31].

Heavy rain and flooding can overwhelm wastewater treatment facilities, contaminating water bodies with pathogenic *E. coli* strains. This is particularly concerning as ETEC is primarily transmitted through contaminated water and is a leading cause of Traveller's diarrhoea in developing regions with poor water sanitation. For instance, ETEC biofilm formation on drinking water contact surfaces in Bangladesh correlated with the warm and humid months, coinciding with epidemic levels of diarrheal disease in impoverished households [32].

Moreover, climate-induced changes in agricultural practices may influence the incidence of *E. coli* pathotypes. Warmer temperatures and altered rainfall can affect the persistence and distribution of *E. coli* on crops, with EAEC and DAEC posing significant risks due to their capacity to adhere to plants. Additionally, using untreated wastewater for irrigation, driven by water scarcity, can introduce pathogenic *E. coli* into the food supply chain, rising public health concerns. Furthermore, it becomes likely that enteropathogenic strains such as EPEC and EIEC, which are commonly associated with person-to-person transmission and foodborne outbreaks, may see altered transmission dynamics due to changes in human behaviour and hygiene practices during extreme weather conditions.

### 2.4 *Listeria monocytogenes (L. monocytogenes)*

*L. monocytogenes* has the ability to multiply at temperatures just above freezing, unlike other bacteria. [33]. Typically, optimal storage at 4 °C slows its growth, often limiting its presence in food to non-harmful levels [34]. Warmer temperatures and higher humidity levels provide favourable conditions for the growth and persistence of *L. monocytogenes* in the natural environment and food industry. For instance, increased temperatures can enhance the persistence and proliferation of *L. monocytogenes* in soil and water, leading to higher contamination levels in crops and livestock [35]. Recent reviewed studies from the USA have highlighted that using surface water for irrigation could be a significant cause of contamination [36]. *L. monocytogenes* was detected in up to 27 % of pond water samples and up to 99 % of wastewater samples from stabilization ponds in the Arctic region of Canada [36].

Temperature may influence the persistence of *L. monocytogenes* in certain regions associated with seafood industry [37]. In this context, temperature plays an important role as it has been shown that L. *monocytogenes* was more likely to be isolated from the springs of Moore region of New York State where air temperatures (10 to 15 °C) matched those in the Salinas region during winter and spring [37]. When contaminated, ready-to-eat foods enable *L. monocytogenes* growth at low temperatures this represents a significant food safety and public health risk [33]. Moreover, temperature fluctuations due to breaks in the cold chain can accelerate the multiplication of these pathogens, causing foods with initially low pathogen levels to exceed safety thresholds in fairly short time periods. Experimental examples in ready-to-eat food matrices have demonstrated that the growth rate of *L. monocytogenes* duplicates as the temperature grows from 4 °C to 8 °C and from 8 °C to 12 °C [33].

Precipitation changes are also affecting the dissemination of *L. monocytogenes*. Heavy rainfall and flooding can lead to the contamination of water sources with L. *monocytogenes*, which has the potential to spread to agricultural fields and food processing plants [38]. In several studies listeriosis outbreaks were linked to waterborne contamination following extreme weather events [39]. Changes in agricultural practices driven by climate change, such as the increased use of untreated wastewater for irrigation due to water scarcity, can introduce *L. monocytogenes* into the food supply and elevated levels of *L. monocytogenes* have been detected in irrigation water from semi-arid regions of South Africa. This would suggest there could be an enhanced risk of human exposure through the consumption of such water. Furthermore, evidence from various African regions indicates excessive precipitation and flooding are related with an elevated risk of *Listeria*-associated foodborne diseases [33,40]. It is hypothesized that conditions concentrate pathogens in limited water sources, increasing the risk of contamination. Analyses from African low-and middle-income countries observed a 5 % increase in the risk of diarrhoea during mild and severe droughts [33,41]. This highlights the need for stringent monitoring and adaptive management strategies to ease the impact of climate changes on food safety.

### 2.5 *Staphylococcus aureus (S. aureus*)

Temperature is instrumental in the survival and spread of *S. aureus*. Higher ambient temperatures, which may further escalate due to climate change, can enhance the pathogen's survival in the environment [42]. For many bacteria the favourable growth temperatures are above 30 °C. Evidence suggests that increased temperatures facilitate plasmid transfer and potentially the gene transfer of resistance genes. Therefore, climate change can directly influence the onset of antibiotic resistance through temperature increases [42]. A study in the United States collected data from 1980 to 2010 on temperature and antibiotic resistance from hospitals and laboratories [[43], [44], [45]] found that antibiotic resistance in three bacterial species (*S. aureus*, *E. coli* and *Klebsiella pneumoniae)* increased with rising local temperatures [[43], [44], [45]]. Similarly, in European countries the impact of climate change on the epidemiology of *Staphylococcus aureus* has been stark and other results have shown that infections inflicted by *S. aureus* occurred more warmer seasons [42]**.** In 2015, researchers estimated higher infection rates with antibiotic-resistant bacteria such as MRSA, third-generation cephalosporin-resistant *E. coli*, carbapenem-resistant *Pseudomonas aeruginosa* and third-generation cephalosporin-resistant *K. pneumoniae* [46]. These findings were confirmed by a 2019 European systematic analysis, which highlighted regional differences in the AMR burden. The rates were higher in Mediterranean countries like Greece and Italy than in Northern European countries, potentially due to climatic differences [46]. For these pathogens, climate related events such as floods, storms, warming, precipitation and droughts were associated with increased antimicrobial resistance [42].

Precipitation patterns and humidity levels also play crucial roles in the survival and transmission of *S. aureus*. Increased humidity can promote the growth of *S. aureus* on surfaces and in the air, potentially leading to higher contamination rates in healthcare settings and community environments [47]. Conversely, acute weather events such as substantial rainfall and floodings can disrupt sanitation infrastructure, leading to increased exposure to such pathogens [42,47]. Hospitals and food sectors should be particularly wary of cockroaches, as they have been found to spread nosocomial pathogens including *S. aureus* and *E. coli* [48]. The geographical proximity of Fuzhou to Taiwan advise that bacterial spreading may frequently occur via environmental vectors such as migratory wildlife and seawater [49].

Farm animals can disseminate *S. aureus* in the environment through air and faeces, contaminated soil, water and crops [50]. The finding of *S. aureus* in surface and air samples backs the idea that farm environments act as carriers of the bacterium. *S. aureus* can become airborne via dust particles [50,51]. Airborne bacteria can reach stable surfaces or can directly assimilated by animals or humans. *S. aureus* carried by livestock and poultry faecal are posing a risk of infection to animals and humans [51].

### 2.6 *Salmonella* spp

Earlier models predicted an increase in *Salmonella* infection rates as ambient temperatures rose, due to climate change, and could lead to around 1000 additional cases annually and a significant increase ($120.000) in healthcare and surveillance costs by 2050. Ambient temperature directly impacts upon the replication rate of *Salmonella*, leading to an increased incidence of disease. The pathogen's ability to survive in dry and dusty conditions makes it capable of persisting in animal feed and low-moisture food processing environments [52]..Other factors such as insufficient aeration, poor ventilation and dust dispersion can favour *Salmonella* persistence in feed and low-moisture food processing plants. This was was previously shown in a study in a feed processing plant, indentifying the intake pit as a key location for contamination [52,53]. However, it can also persist in high-moisture food environments involving eggs, meat, and poultry [52] and global warming is predicted to encourage the colonisation and growth of Salmonella in broiler flocks [54].

The influence of ambient temperature on *Salmonella* development at various stages of the food chain is well-documented. This includes bacterial contamination during transport, raw food production and improper storage [15]. The optimal temperature for *Salmonella* growth is between 35 °C and 37 °C, with growth significantly inhibited at 15 °C [8]. Previous estimations have shown that a 1 °C increase in the average weekly maximum temperature can lead to an 8.8 % increase in weekly salmonellosis incidences [15,55]. A study identified that a 1 °C increase in the average weekly minimum temperature is associated with a 5.8 % increase weekly in the number of cases [15,55]. *Salmonella* survival in soils, animal effluents and surface waters is reduced at higher temperatures and during fluctuations of ambient temperature or freeze-thawing circumstances [13]. In contrast, *Salmonella* is detected in poultry and oyster samples more frequently at increased temperatures [13].The consequence of temperature on the survival and growth is not uniform across all environments. In the absence of proper surveillance strategies, monitoring, and disinfection, prolonged increased temperatures can lead to an increase in the counts of *Salmonella* cells in poultry flocks [54]. Based on the research, there is a clear need for targeted interventions to address the specific challenges posed by climate change to different sectors of the food chain.

In the Republic of Ireland, climate changes are expected to increase the incidence of salmonellosis by 2 % and campylobacteriosis by 3 % [56]. The seasonality of *Salmonella* is a direct result of its sensitivity to temperature. Almost all salmonellosis cases in Europe are reported during the summer months, with the incidence of *Salmonella* typically reduced in colder, northern countries compared to those with warmer climates [13,15]. In 2017, the WHO reported that in Europe, climate change caused a 5–10 % increase in salmonellosis cases for each 1 °C rise in weekly temperatures when ambient temperatures surpassed 5 °C [10,57]. Additionally, a study completed in Kazakhstan found that salmonellosis cases has the potential to rise to 5.5 million with a 1 °C increase in the average monthly temperature [57]. In New Zealand, the infection risk of salmonellosis is related with moderately high temperatures in Auckland and Christchurch, yet interestingly no significant association between temperature and salmonellosis risk was found in Wellington, which is further south and has lower ambient temperatures [13]. To underline the importance of the temperature, a study conducted in New York, USA, demonstrated inconsistent results or no relationship between the incidence of *Salmonella* and variables such as precipitation and humidity - only ambient temperature impacted upon Salmonella infections [13,58]. Data from Maryland observed a 4.1 % increase in salmonellosis risk due to temperature increase and a 5.6 % increase due to increase in precipitation with higher risks posed in coastal communities compared to non-coastal ones [13,59]. In conclusion, the Mississippi study identified a significant positive correlation between temperatures and *Salmonella* infections, with small temperature rises related to four *Salmonella*-associated cases [60]. Anticipating changes in salmonellosis patterns due to climate change is challenging because of the inherent complexity and uncertainty surrounding the effects on the host-agent-environment interactions. Additionally, methodological issues, such as the high correlation between climatic variables, complicate the identification of true explanatory factors and risk prediction [13].

## 3 Conclusions

Most of the available literature points towards a rise in climate-associated foodborne pathogen associated outbreaks across the globe due to the varying responses of different serovars and in their transmission patterns to ambient temperature. Future One Health projections will help to mitigate the impact of climate change on the spread of foodborne pathogens which should differentiate between specific virulence factors affected and transmission mechanisms.

## Funding resources

None.

## CRediT authorship contribution statement

**Igori Balta:** Writing – review & editing, Writing – original draft, Conceptualization. **Joanne Lemon:** Writing – review & editing, Writing – original draft, Conceptualization. **Ciara Murnane:** Writing – review & editing, Writing – original draft. **Ioan Pet:** Writing – review & editing, Writing – original draft, Conceptualization. **Teodor Vintila:** Writing – review & editing, Writing – original draft, Conceptualization. **David McCleery:** Writing – review & editing, Writing – original draft, Conceptualization. **Todd Callaway:** Writing – review & editing, Writing – original draft, Conceptualization. **Alastair Douglas:** Writing – review & editing, Writing – original draft, Conceptualization. **Lavinia Stef:** Writing – review & editing, Writing – original draft, Conceptualization. **Nicolae Corcionivoschi:** Writing – review & editing, Writing – original draft, Conceptualization.

## Declaration of competing interest

The authors declare that they have no known competing financial interests or personal relationships that could have appeared to influence the work reported in this paper.

## Data Availability

No data was used for the research described in the article.
